# Current and future burden from Lyme disease in Québec as a result of climate change

**DOI:** 10.14745/ccdr.v49i10a06

**Published:** 2023-10-01

**Authors:** Marion Ripoche, Alexandra Irace-Cima, Ariane Adam-Poupart, Geneviève Baron, Catherine Bouchard, Alex Carignan, François Milord, Najwa Ouhoummane, Pierre A Pilon, Karine Thivierge, Kate Zinszer, Diane Chaumont

**Affiliations:** 1 Institut national de la santé publique du Québec, Montréal, QC; 2 Direction de la santé publique de l’Estrie [Estrie Public Health Department], CIUSSS de l’Estrie-CHUS, Sherbrooke, QC; 3 Public Health Agency of Canada, Saint-Hyacinthe, QC; 4 Université de Sherbrooke, Sherbrooke, QC; 5 Direction de Santé Publique de la Montérégie [Montérégie Public Health Department], CISSS de la Montérégie-Centre, Longueuil, QC; 6 Direction de Santé Publique de Montréal [Montréal Public Health Department], Montréal, QC; 7 Université de Montréal, Montréal, QC; 8 Ouranos, Montréal, QC

**Keywords:** tick-borne illness, Lyme disease, *Borrelia burgdorferi*, climate change, burden

## Abstract

**Context:**

Environmental changes will foster the spread of *Ixodes scapularis* ticks and increase the incidence of Lyme disease in Québec in the coming years. The objective of this study is to estimate the epidemiological and clinical burden and part of the current economic burden of Lyme disease in Québec and to estimate the number of cases expected by 2050.

**Methods:**

Cases of Lyme disease reported in Québec from 2015 to 2019 were used to describe their demographic, geographical and clinical characteristics and the cost of their initial care. Three incidence rate scenarios were then developed to estimate the number of cases expected by 2050, based on demographic and climate projections.

**Results:**

From 2016 to 2019, 1,473 cases of Lyme disease were reported in Québec. Over 90% of those cases were acquired in two regions of southern Québec (Estrie and Montérégie), while the individuals infected were residents from all over Québec. The average age of cases is 44 years and 66% of infections were at the localized stage, the first stage of Lyme disease. The cost of initial care is estimated at an average of $182 CAN per patient ($47 CAN at the localized stage and $443 CAN at the disseminated stage). According to projections, over 95% of the Québec population will live in a climate zone conducive to the establishment of ticks by 2050, with a number of cases acquired in Québec being 1.3 to 14.5 times higher than in 2019, depending on the incidence rate scenario used.

**Conclusion:**

The epidemiological burden is concentrated primarily in southern Québec, but the clinical and economic burden is already distributed throughout the province. The projections for 2050 should help the regions of Québec adapt and optimize public health protection measures.

## Introduction

Cases of Lyme disease have been on the rise for several years in Québec, as in the rest of Canada ((1)). This trend is expected to continue with expected climate and environmental changes ((2)). However, the burden of Lyme disease, namely its epidemiological, clinical and economic characteristics ((3)), is still poorly documented in Québec.

Lyme disease, caused by the bacterium *Borrelia burgdorferi*, is transmitted by the *Ixodes scapularis* tick in eastern North America. The infection evolves in three clinical stages: the localized stage, characterized by erythema migrans; the early disseminated stage, with systemic, neurological or cardiac symptoms; and the late disseminated stage, characterized primarily by Lyme arthritis ((4)). Rising temperatures linked to climate and environmental change are expected to favour the survival of tick populations, extend tick activity over the year and foster the establishment of tick populations in new geographic areas, at the same time as there is an increase in the distribution area of hosts such as the white-tailed deer or white footed mice ((2)). As a result, the season and zone for human exposure to ticks, and thus the incidence of Lyme disease in Québec, is expected to increase over time.

Lyme disease has been a notifiable disease (ND) in Québec since 2003 ((1)). The demographic and geographic characteristics of cases are published annually by the *Institut national de santé publique du Québec and the Ministère de la Santé et des Services sociaux* ((1,5)). Several studies have described the clinical picture of Lyme disease, but only for some regions of Québec ((6–14)). Some costs associated with Lyme disease have been assessed in Ontario ((15)) and the United States ((16–20)), but their results cannot be transposed directly to Québec due to differences in healthcare systems. To our knowledge, only two studies have estimated the number of cases and the anticipated costs based on climate change ((21,22). The most recent study, conducted by the Canadian Institute for Climate Choices (CICC), estimates that there will be 8,500 new cases of Lyme disease each year in Canada by the middle of the century (3,000 in Québec), for an annual cost of $3M in health expenditures ((22)). However, those studies are not based on surveillance data from Québec, which limits their interpretation.

Our study describes the current burden of Lyme disease in Québec, from an epidemiological and clinical perspective, based on human surveillance data. Exposure to ticks was also considered to have a broader view of the burden for the province, based on acarological surveillance data. Moreover, to add an economic dimension to the burden, the direct cost to the healthcare system for initial care and hospitalization of cases was calculated. Finally, the number of expected cases by 2050 was also estimated, taking into account various demographic, climate and incidence rate scenarios.

## Methods

### Data source

#### Human cases of Lyme disease

Human cases of Lyme disease reported in Québec by physicians or laboratories between January 1, 2015, and December 31, 2019, were extracted from the registry of NDs ((1)). As the ND does not include clinical data for our study period, that data was found in the reports available from the public health departments (Direction de santé publique [DSPu]) in Estrie (n=105 cases in 2017), Montérégie (n=231 cases in 2016–2018) and Montréal (n=69 cases in 2016–2017). These reports present some results from epidemiological investigations of cases reported between 2016 and 2018 ((8–11,23,24)), such as stage and clinical signs, proportion of cases hospitalized and length of hospitalization. Data published by Musonera *et al.*, ((14)), analyzing the medical records of cases reported and treated at a hospital in Estrie and Montérégie between 2004 and 2017 (n=272), were also considered in estimating some clinical criteria not available in the ND (proportion of cases by clinical stage, proportion of cases hospitalized and length of hospitalization).

#### Exposure to *Ixodes scapularis* ticks

Data on the people who reported an *I. scapularis* tick in Québec between January 1, 2015, and December 31, 2019, is from the passive acarological surveillance program managed by the *Laboratoire de santé publique du Québec* ((1)). That laboratory receives and identifies ticks collected from humans that are voluntarily submitted by physicians. The *I. scapularis* ticks are then sent to the National Microbiology Laboratory to check for the presence of *B. burgdorferi* and other pathogens ((1)). To be sure of the tick exposure location, people who have travelled outside their home municipality in the 14 days prior to the bite and those whose travel history was unknown were excluded from the geographic analyses.

#### Cost of initial care and hospitalization

Only the direct costs to the healthcare system, i.e. the cost of initial care for the case (consultation and treatment) and hospitalizations, were considered in this study. The cost of initial care of a case is based on the cost of medical consultations in Québec published by the *Régie de l’assurance maladie du Québec* (RAMQ) ((25,26)) and the cost of initial treatment published by the *Institut national d’excellence en santé et en services sociaux* (INESSS) ((27)). The cost of hospitalizations is based on data for Québec from the Canadian Institute for Health Information ((28)).

#### Demographic projections

The current population of Québec was estimated based on the 2016 census ((29)). The projections are from the demographic trends published by the *Institut de la statistique du Québec*, with a moderate scenario, a low scenario and a high scenario, to estimate the possible evolution of Québec’s population by 2050 ((30)).

#### Climate projections

Temperature is a significant factor in the establishment of tick populations ((31–39)). The threshold of 2,800 degree-days (dd) >0°C over a year was validated in several studies as an indicator of areas where *I. scapularis* ticks can survive in Québec ((32,40)) and was used as an indicator in our study. The annual accumulation of >0°C dd between 2009 and 2100 (average: over 30 years) for all of Québec (10 km x 10 km grid) is from the Climate Data portal ((41)), for greenhouse gas emission scenarios RCP 4.5 (moderate emissions scenario) and RCP 8.5 (high emissions scenario).

## Analyses

### Epidemiological and clinical portrait

The epidemiological portrait looked at all cases of Lyme disease reported in Québec in the ND and all persons who reported a tick to the surveillance program between 2015 and 2019, that is, the number of cases by age, sex, likely region of acquisition or exposure to ticks, region of residence and month in which the first symptoms appeared or of exposure to ticks.

To prepare a clinical portrait of cases reported during our study period, the data published by the DSPu ((8–11,13,23,24)) and by Musonera *et al.* ((14,42)) for the Estrie, Montérégie and Montréal regions were used. The average percentages of cases by stage and clinical signs was estimated based on these data, and the percentages were related to all cases reported in Québec between 2015 and 2019 to estimate the number of cases by stage and clinical signs during our study period. Chi-squared tests (*p-*value=0.05) were conducted in R software (R version 4.0.2) to compare categorical variables. The cases reported and the persons who reported a tick to passive surveillance were mapped by likely region of acquisition or exposure using QGIS (version 3.14.1).

### Cost of initial care and hospitalization

The cost of care was calculated by reported case and by clinical stage. Initial care includes the first medical consultation with a physician and the initial treatment prescribed based on the clinical signs. At the localized stage, a consultation with a general practitioner is recorded, while consultations are recorded at the disseminated stage with a general practitioner and for a visit and follow-up with a specialist. The initial treatment considered is the treatment recommended by INESSS ((27)). Two studies indicate that the prescribed treatment in Québec is appropriate in over 85% of cases ((14,24)). The cost of hospitalizations was estimated separately taking into account the average length and the cost of a stay.

### Projected number of cases expected by 2050

All municipalities in Québec were ranked as favourable or unfavourable to the establishment of ticks based on the threshold of 2,800 dd in 2019 and by 2050, to estimate the favourable area for establishment of tick populations and its growth over time. Degree-days were calculated for each municipality by determining the average dds in the area based on observations in 2019 (average: 2015–2019) and based on projections for 2050 (average: 2014–2071) for RCPs 4.5 and 8.5.

The average incidence rates were then calculated in the area favourable to the establishment of ticks (>2,800 dd) and outside that area (<2,800 dd) for the reference year 2019 (year with the highest incidence rate in our study period) as follows: number of cases reported in the area/number of residents in the area. Finally, three incidence rate scenarios were considered to account for the possible evolution by 2050: Scenario 1 (stable incidence rate): the incidence rates remain similar to those calculated in 2019 inside and outside the area favourable to the establishment of ticks; Scenario 2 (higher incidence rate in one region): the incidence rates remain similar to those calculated in 2019, except in the Estrie region, which is the region with the highest incidence rate in 2015–2019; for that region, the incidence rate calculated in that region in 2019 is used; Scenario 3 (high incidence rates): the incidence rate in Estrie calculated in 2019 is applied to all areas favourable to the establishment of ticks by 2050. These incidence rates made it possible to calculate the number of cases expected based on demographic projections for Québec. Finally, the analyses conducted combine two climate scenarios (RCP 4.5 and RCP 8.5), three demographic scenarios (moderate, low and high) and three incidence rate scenarios (stable, higher in one region, high) for a total of 18 scenarios. The direct costs for healthcare expenditures for the 2050 horizon were calculated by correlating the number of cases expected in 2050 to the cost per patient estimated in 2019.

## Results

### Epidemiology

#### Incidence rate and demographic characteristics

Between 2015 and 2019, 1,473 cases of Lyme disease were reported in Québec, giving an average incidence rate of 3.58 cases/100,000 inhabitants for the period for the entire province. Men represented 58% of cases reported and the average age is 44 years (range: <1 year–89 years, median: 48 years) (**Table 1**). The distribution by age is bimodal: 0 to 9 years represents 10% of cases and 50 to 69 years represents 39% of cases. Distribution by age group is similar among men and women (*p*=0.35).

**Table 1:** Epidemiological characteristics of cases of Lyme disease reported in Québec, 2015–2019

Reported cases	n	%
**Number of cases (n=1,473)**		
Acquired in Québec	1,098	74%
Acquired outside Québec	334	23%
Unknown	41	3%
**Age group (years) (n=1,473)**		
0–9	144	10%
10–19	139	9%
20–29	96	7%
30–39	171	12%
40–49	198	13%
50–59	281	19%
60–69	300	20%
70–79	126	9%
80–89	18	1%
**Sex (n=1,469)**		
Male	858	58%
Female	611	42%
**Likely location of acquisition outside Québec (n=334)**		
United States	174	52%
Other province of Canada	94	28%
Europe	49	15%
Other	17	5%
**Likely location of acquisition inside Québec (n=1,025)**		
Estrie	590	58%
Montérégie	352	34%
Mauricie-et-Centre-du-Québec, Outaouais, Lanaudière, Laurentides, Laval, Montréal	78	8%
Capitale-Nationale, Chaudière-Appalaches, Saguenay–Lac-Saint-Jean, Côte-Nord	5	<1%
Abitibi-Témiscamingue, Gaspésie–Îles-de-la-Madeleine, Bas-Saint-Laurent, Nord-du-Québec	0	0%
**Cases acquired in Québec in a known region (n=1,025)**		
Acquired in region of residence	875	85%
**Region of residence (n=1,473)**		
Estrie	548	37%
Montérégie	436	29%
Montréal	229	16%
Mauricie et Centre-du-Québec, Outaouais, Lanaudière, Laurentides, Laval	230	16%
Capitale-Nationale, Chaudière-Appalaches, Saguenay–Lac-Saint-Jean, Bas-Saint-Laurent	21	1%
Abitibi-Témiscamingue, Gaspésie–Îles-de-la-Madeleine, Côte-Nord, Nord du Québec	9	<1%

The demographic characteristics are similar for the 6,392 individuals who reported ticks to the passive surveillance program between 2015 and 2019. Of those individuals, 57% were men and the average age was 39 years (range: <1 year to 93 years, median 42 years). The distribution by age is bimodal: 0 to 9 years represents 18% of cases and 50 to 69 years represents 35% of cases. This distribution is similar among men and women (*p*=0.08).

#### Likely region of acquisition or exposure

**Figure 1:**
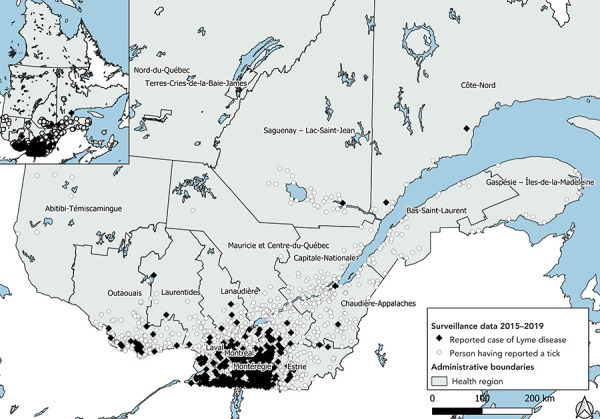
Likely location of acquisition of reported cases of Lyme disease and exposure of persons who reported a tick, Québec, 2015–2019

A total of 74% of reported cases acquired their infection in Québec, primarily in the south of the province: 58% in Estrie and 34% in Montérégie (Table 1 and **Figure 1**). The incidence rate is below 6 cases/100,000 inhabitants for all regions of Québec, except Estrie, which averaged 35 cases/100,000 inhabitants for 2015–2019. Despite a large number of cases, the incidence rate in Montérégie is relatively low (5 cases/100,000 inhabitants), mainly due to the size of the region’s population. Of the cases acquired outside Québec, 52% were acquired in the United States (state not specified).

The geographic distribution of individuals who reported a tick to the passive surveillance program is larger than that of acquisition of Lyme disease (Figure 1). People reported ticks in all regions of Québec, except Nord-du-Québec. Terres-Cries-de-la-Baie-James and Nunavik. More people reported ticks in southern Québec (29% in Estrie, 23% in Montérégie and 13% in Outaouais).

### Region of residence

The region of residence of the case may be different from the region where the disease was acquired. Between 2015 and 2019, Lyme disease affected residents in all regions of Québec, except Nunavik and Terres-Cries-de-la-Baies-James. Of the reported cases, 37% lived in Estrie, 30% in Montérégie and 16% in Montréal (Table 1). The other regions account for less than 60 cases among their residents.

On average, cases acquired in the person’s region of residence represent 85% of cases reported and acquired in Québec (59% of all reported cases), but there continue to be significant variations between regions. For Montérégie and Estrie, most cases are acquired in their region of residence (73% and 90% respectively), while that figure is only 1% for Montréal. Most cases reported in Montréal are acquired in another region of Québec (40%, mostly in Estrie and Montérégie) or outside Québec (53%).

### Seasonality

**Figure 2:**
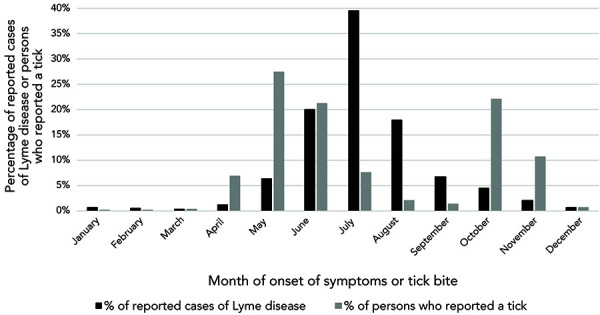
Month of onset of symptoms in reported cases of Lyme disease and reported tick bites, Québec, 2015–2019

Cases of Lyme disease can occur throughout the year. However, in at least three of four cases (77%), the onset of symptoms is between June and August, with a peak in July (39% of cases) (**Figure 2**).

Ticks are also reported to the surveillance program throughout the year, with 60% of people reporting between April and July (peak in May) and 35% between October and November (peak in October) (Figure 2). People mainly reported adult ticks (92% of ticks reported), and 19% of ticks analyzed were infected with *B. burgdorferi*.

## Clinical characteristics

### Reporting of cases

Over the period of 2015–2019, cases were reported on average 60 days after the onset of the first symptoms (median: 36 days; standard deviation: 124 days). Only 32% of cases were reported directly by a physician and not by a laboratory following a diagnostic test (**Table 2**).

**Table 2:** Clinical characteristics of cases of Lyme disease reported in Québec, 2015–2019

Cases reported 2015–2019	n	%
**Time between onset of symptoms and reporting of the case (n=1,329)^a^**		
<1 month	609	46%
1–3 months	564	42%
>3 months	156	12%
**Case reported (n=1,473)^a^**		
By a physician	477	32%
By a laboratory	996	68%
**Clinical stages (n=1,473)^a^**		
Localized	972	66%
Disseminated	501	34%
Early	398	27%
Late	103	7%
**Clinical signs (n=1,473)^b,c^**		
**General**		
Fever	427	29%
Fatigue	501	34%
Headache	412	28%
**Cutaneous**		
Typical erythema migrans	957	65%
Multiple erythema migrans	324	22%
Acrodermatitis chronica atrophicans	0	0%
**Musculoskeletal**		
Myalgia	368	25%
Arthralgia	427	29%
Arthritis	162	11%
**Neurological**		
Stiff neck	162	11%
Facial paralysis	118	8%
Radiculopathy	15	1%
Meningitis	74	5%
**Cardiac**		
Heart rate disorder	15	1%
Atrioventricular block (AV block)^d^	29	2%
Carditis	15	1%
**Evolution at time of investigation (n=1,473)^a^**		
Recovery	1 046	71%
After-effects	20	1%
Unknown	407	28%
**Hospitalization (n=1,473)^b^**		
1–4 days	103	7%

^a^ Estimation of the number and percentage based on the register of notifiable diseases (ND) (n=1,473)

^b^ Estimation of the percentage based on data available from public health departments (DSPu) (8–11,23,24) and Musonera *et al.* (14,42), and extrapolation to 1,473 cases to estimate the number of cases per stage and clinical sign and the number of hospitalizations

^c^ One person can present several symptoms

^d^ Atrioventricular blocks (AV blocks): 0.6% of 1^st^ degree AV blocks, 1% 2^nd^ and 3^rd^ degree AV blocks

### Clinical signs and stages

To prepare a clinical portrait of the 1,473 cases, the data provided by the DSPu (n=405 cases) and Musonera *et al.*, ((14)) (n=272 cases) were used. On average, 66% of cases of Lyme disease are at the localized stage, and 34% at the disseminated stage when reported (Table 2).

For all cases, 65% present typical erythema migrans and 22% multiple erythema. The most commonly cited general symptoms are fatigue (34%), fever (29%), arthralgia (29%), headaches (28%) and myalgia (25%). There were neurological symptoms in 25% of cases, cardiac symptoms in 3% of cases and Lyme arthritis in 11% of cases (Table 2). One person can present multiple symptoms.

### Hospitalization

According to data reported by the DSPu and Musonera *et al.*, ((14), an average of 7% of reported cases required short-term hospitalization (1–4 days), or 103 cases over our study period.

### Evolution and death

The ND indicates recovery (improvement or disappearance of clinical signs) at the time of the epidemiological investigation for 71% of cases and after-effects for 1% of cases. No deaths were reported in the ND for our study period.

## Cost of initial care and hospitalization

### Initial care

For a case at the localized stage, the cost of initial care (consultation and treatment) is estimated at $47 CAN ($31–$63 CAN). For a case at the disseminated stage, that cost is estimated at $443 CAN ($172–$714 CAN depending on the clinical signs). Applied to all cases, initial care costs an average of $182 CAN per case ($979–$284 CAN) (**Table 3**).

**Table 3:** Cost of initial care of reported cases of Lyme disease by clinical stage, Québec, 2015–2019

Québec, 2015–2019	n	Cost per case (CAN$)			Cost 2015–2019 (CAN$)			Cost per year (CAN$)		
		Average	Min	Max	Average	Min	Max	Average	Min	Max
**Localized stage**	**972;** **(66%)**	**46.90**	**31.14**	**62.66**	**45,586.80;** **(17%)**	**30,268.08**	**60,905.52**	**9,117.36**	**6,053,61**	**12,181.10**
General practitioner consultation^a^	972	32.84	19.42	46.25	31,920.48	18,876.24	44,955.00	6,384.10	3,775.25	8,991.00
Doxycycline 200 mg, 1/day, 10–14 days^b^	972	14.07	11.72	16.41	13,676.04	11,391.84	15,950.52	2,735.21	2,278.37	3,190.10
**Disseminated stage**	**501;** **(34%)**	**443.17**	**172.08**	**714.25**	**222,028.17;** **(83%)**	**86,212.08**	**357,839.25**	**44,405.63**	**17,242.41**	**71,567.85**
General practitioner consultation^a^	501	32.84	19.42	46.25	16,452.84	9,729.42	23,171.25	3,290.57	1,945.88	4,634.25
Consultation + specialist follow-up^a^	501	148.76	136.25	161.27	74,528.76	68,261.25	80,796.27	14,905.75	13,652.25	16,159.25
Treatment according to clinical signs^b^	501	261.57	16.41	506.73	131,046.57	8,221.41	253,871.73	26,209.31	1,644.28	50,774.35
**Total 2015–2019**	**1,473;** **(100%)**	**181.63**	**79.05**	**284.20**	**267,540.99;** **(100%)**	**116,440.65**	**418,626.60**	**53,508.19**	**23,288.13**	**83,725.32**
Hospitalization	103; (7%)	2,000.00	1,000.00	4,000.00	206,000.00	103,000.00	412,000.00	41,200.00	20,600.00	82,400.00

Abbreviations: N, number; Min, minimum, Max, maximum

^a^ According to the *Régie de l’assurance maladie du Québec* (RAMQ) (25,26)

^b^ According to the *Institut national d’excellence en santé et en services sociaux* (INESSS) (27)

For the 1,473 cases reported during the period of 2015–2019, the cost would be $267,541 CAN ($116,440–$418,627 CAN) over 5 years, or $53,508 CAN per year ($23,288–$83,725 CAN). Cases at the disseminated stage represent 34% of cases reported, but 83% of the cost of treating cases (Table 3).

### Hospitalization

The estimated 103 hospitalizations represent a cost of $589,200 CAN for 2015–2019, or an average of $117,840 CAN/year (Table 3).

### Projections for 2050

Québec’s population is expected to increase from approximately 8,460,000 in 2019 to approximately 9,550,000 by 2050, an average increase of 13% based on the moderate demographic scenario (low scenario: 8,230,000 inhabitants [-3%], high scenario: 10,8550 000 inhabitants [+28%]) (**Table 4**).

**Table 4:** Number of reported cases of Lyme disease based on the various demographic, climate and incidence rate scenarios, Québec, 2019 and 2050

Scenarios	2019	Emission and demographic scenarios for 2050					
		RCP 4.5			RCP 8.5		
		Moderate	Low	High	Moderate	Low	High
**Human population**							
Québec	8,460,000	9,550,000	8,230,000	10,850,000	9,550,000	8,230,000	10,850,000
Area dd >2,800	6,187,253	9,177,134	7,908,672	10,426,378	9,350,074	8,057,708	10,622,859
Percentage of Québec population	73%	96%	96%	96%	98%	98%	98%
**Incidence scenario^a^**							
**Scenario 1**							
Number of cases acquired in Québec	381	494	426	562	499	430	567
Increase from 2019	+0%	+30%	+12%	+47%	+31%	+13%	+49%
**Scenario 2**							
Number of cases acquired in Québec	381	693	609	781	698	613	787
Increase from 2019	+0%	+82%	+60%	+105%	+83%	+61%	+107%
**Scenario 3**							
Number of cases acquired in Québec	381	5,535	4,770	6,289	5,635	4,856	6,403
Increase from 2019	+0%	+1,353%	+1,152%	+1,551%	+1,379%	+1,175%	+1,580%

Abbreviations: dd, degree-days >0^0^C; RCP, Representative Concentration Pathway

^a^ Incidence scenario: Scenario 1: Incidence rates similar to the moderate rates in 2019, i.e. 5.29 cases/100,000 inhabitants in the area with dd >2,800 and 2.39 in the area with dd <2,800; Scenario 2: incidence rates similar to the moderate rates in 2019 except in Estrie, which maintains its incidence rate from 2019, i.e. 60.22 cases/100,000 inhabitants in Estrie; 5.29 cases/100,000 inhabitants in the area with dd >2,800 and 2.39 in the area with dd <2,800; Scenario 3: incidence rates similar to the Estrie rates in 2019, i.e. 60.22 cases/100,000 inhabitants in the area dd >2,800 and 2.39 in the area dd <2,800

**Figure 3:**
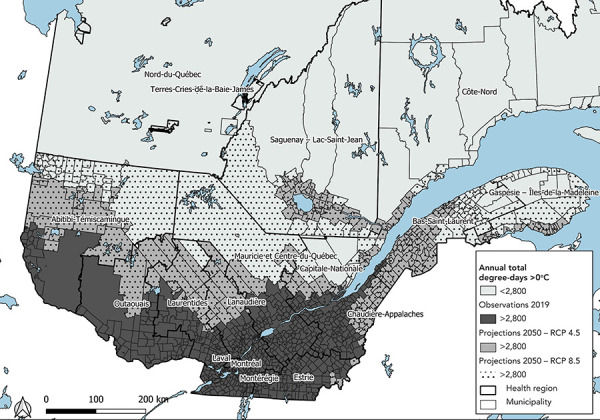
Municipalities located in the climate zone favourable to the establishment of ticks based on the climate scenarios, Québec, 2019 and 2050 Abbreviation: RCP, Representative Concentration Pathway

**Figure 3** shows that the current climate limits the extent of the area favourable to the establishment of ticks in the southernmost part of Québec. With rising temperatures resulting from increased greenhouse gas emissions, the climate is becoming favourable in almost all inhabited areas in Québec. By 2050, 96% to 98% (RCP 4.5 and 8.5) of Québec’s population will live in the climate zone favourable to the establishment of tick populations, compared with 73% in 2019 (Table 4 and Figure 3).

In 2019, the incidence rate in the area favourable to ticks (dd >2,800) is estimated at 5.29 cases/100,000 inhabitants and at 2.39 cases/100,000 inhabitants outside that area (dd <2,800). In scenario 1—with incidence rates similar to those in 2019—projections for 2050 suggest an increase of about 30% in the number of cases acquired in Québec compared with 2019, with the moderate demographic scenario and RCP 4.5, i.e. 494 cases expected compared with 381 in 2019 (426 cases, +12% and 562 cases, +47% for the low and high demographic scenarios). In scenario 2—considering higher incidence rates in the Estrie region, i.e. 60.22 cases/100,000 inhabitants in 2019—the number of cases would almost double compared with 2019, with 693 cases expected for 2015 (609 to 781 cases). In Scenario 3, the entire area favourable to ticks would have an incidence rate similar to that of Estrie, i.e. 60.22 cases/100,000 inhabitants, and the rest of the province would have a rate of 2.39 cases/100,000 inhabitants. By 2050, the number of cases would be 14 times higher than in 2019, with 5,535 cases expected (4,770 to 6,289 cases). For all three scenarios, the projections are relatively similar under RCP 8.5 (Table 4 and Figure 3).

The 494 to 5,535 cases would represent a cost of $88,090 to $1,005,322 CAN by 2050 for initial care. The cost of hospitalization of the 35 to 387 cases would be $140,000 to $1,548,000 CAN. The cost of cases acquired outside Québec (currently 23% of reported cases) would be added to these results.

## Discussion

This study describes the current burden of Lyme disease in Québec from an epidemiological, clinical and, in part, economic perspective, based on human and acarological surveillance data from Québec for the period of 2015–2019. It also estimates the number of expected cases by 2050, considering various demographic, climatic and incidence rate scenarios.

The overall incidence rate in Québec is 3.58 cases reported/100,000 inhabitants for the period of 2015–2019. In 2019, Québec had the third highest number cases of Lyme disease among Canadian provinces, behind Ontario and Nova Scotia (43). The incidence rate is higher in the southern parts of Québec, where the disease is endemic, than it is in the other parts of the province, where it is not yet present or is emerging (1,5). The demographic and seasonality characteristics are similar to those for Canada as a whole (43,44), Ontario (45) and Nova Scotia (46).

The epidemiological burden is concentrated in a few regions in southern Québec, where the disease is endemic, but the clinical and economic burden concerns all regions of Québec. Indeed, cases are reported in all regions of Québec and are managed *a priori* by the healthcare system in their region of residence, whether or not the infection was acquired there. In addition, tick exposure is possible in much of the province, even though the majority of exposures are reported in Estrie and Montérégie, two regions where tick populations have been known to be present for over 10 years (1).

Most cases are reported, and thus diagnosed, at the localized stage of the disease. As a result, 65% of reported cases presented erythema migrans, an early symptom of the disease, and only 11% presented Lyme arthritis, the most advanced stage of the disease. Other Canadian studies report a similar proportion of erythema migrans and neurological and cardiac symptoms, but more cases of arthritis (43–47). However, care must be taken in interpreting the results, as access to clinical data is difficult and often limited to the regions most affected by Lyme disease, which limits extrapolation to all of Québec. In addition, the clinical signs of Lyme disease are often not very specific and the stages are hard to determine in practice or from medical records (4,27).

The average cost of initial care is estimated at $182 per patient and varies widely depending on the clinical signs ($47 on average for typical erythema migrans, $443 for carditis). These costs are based on recommendations for the initial treatment of cases (4) and do not consider the extension of treatment in some cases. In Québec, however, clinical evolution is favourable in 99% of serious cases, with objective clinical signs disappearing in less than three months (14,42). In addition, a study conducted in Ontario estimates that most costs occur within 30 days of diagnosis (15). The Canadian Institute for Climate Choices (CICC) (22,48) estimates the long-term cost of Lyme disease (hospitalization, outpatient medical care, medication, treatment and lost productivity) at an average of $26,795 CAN per case in 2016. The authors state that 97% of costs are related to a loss of quality of life and only 0.9% to direct costs of healthcare expenditures, or an average of $241 CAN per case (including hospitalization, medical care and treatment), which is consistent with our study and explains the significant differences between studies of the economic burden of Lyme disease, depending on the costs considered.

Demographic and climate projections suggest 1.3 to 14.5 times more cases acquired in Québec in 2050 than in 2019, with about 500 to 5,500 cases expected by 2050, depending on the incidence rate scenarios. The increased number of cases seems to be related more to the evolution of the incidence rate than the progression of ticks in the area as a result of climate change. In fact, there is little difference between the RCP 4.5 and 8.5 emission scenarios, as human population growth and sprawl are still limited in Québec: the northern parts of the province are sparsely populated, and 80% of the human population lives along the St. Lawrence River or in the regions south of the river (49), which are already areas where Lyme disease is endemic or areas favourable to the establishment of ticks. Nevertheless, the regions will probably not be affected by Lyme disease in the same way. Locally, some municipalities will probably have a higher incidence rate than others, depending on the combination of demographic growth and the increase in tick density in their area. Thus, simply having a region with a higher incidence rate than the rest of the province (Scenario 2) almost doubles the number of cases expected in 2050.

The complexity of the biological models yields different results depending on the parameters chose in the studies (21,22,48,50). The consequences of higher temperatures on the impact of Lyme disease are hard to assess, as the relationship is probably not linear (2,37,50). Other factors will also play a role in the progression of Lyme disease in Québec, such as changes in habitat and the host community favourable to ticks, increased outdoor human activity, urbanization of areas where the disease is endemic, and awareness among the general public of adopting preventive measures (2). Similarly, the evolution of cases acquired outside Québec remains difficult to estimate. Beyond the expected number of cases, it is the general trend that must be considered in adaptation plans, with an increase in the number of cases and geographic distribution, thus impacting regions and human populations that are not yet affected much by the disease.

## Limitations

There are several limitations to surveillance data on Lyme disease. First, the number of cases reported or diagnosed does not represent the actual number of cases of Lyme disease (51), which has an impact on the estimation of the burden and related projections. Similarly, the number of people who reported a tick to Québec’s passive surveillance system underestimates the actual number of people bitten by a tick (51).

The clinical burden is based on epidemiological investigations conducted in regions where Lyme disease has been endemic for several years, which may limit the validity of their extrapolation to other regions of Québec. More detailed clinical studies of all cases of Lyme disease in Québec would be needed to refine the clinical picture.

The economic estimate presented in this study does not take into account all costs associated with Lyme disease. For example, some costs, such as absenteeism from work, reduced quality of life, the cost of laboratory tests, post-exposure prophylaxis or disease surveillance, were not considered but contribute to the total burden of Lyme disease in Québec.

## Conclusion

This study provides an initial portrait of the burden of Lyme disease in Québec. Although the cases are acquired primarily in the southern part of the province, all of Québec is already concerned about the management of Lyme disease. The results present an order of magnitude of the current and future burden of Lyme disease, how to prepare the regions of Québec to adapt and optimize public health protection measures.
